# Speech in noise prediction by use of cortical auditory evoked potentials in normal hearing and sensorineural hearing loss: a systematic review

**DOI:** 10.3389/fnins.2026.1713335

**Published:** 2026-04-09

**Authors:** Lana Biot, Emilie Cardon, Laura Jacxsens, Annick Gilles, Vincent Van Rompaey, Marc J. W. Lammers

**Affiliations:** 1 Resonant Labs Antwerp, Department of Translational Neurosciences, Faculty of Medicine and Health Sciences, University of Antwerp, Wilrijk, Belgium; 2 Department of Otorhinolaryngology, Head and Neck Surgery, Antwerp University Hospital (UZA), Edegem, Belgium; 3 Department of Rehabilitation Sciences and Physiotherapy, Faculty of Medicine and Health Sciences, University of Antwerp, Wilrijk, Belgium; 4 Department of Education, Health and Social Work, University College Ghent, Ghent, Belgium

**Keywords:** cortical auditory evoked potentials, non-task related, sensorineural hearing loss, speech perception, systematic review

## Abstract

**Introduction:**

Speech perception in noise (SPiN) is a critical challenge for individuals with sensorineural hearing loss (SNHL), and current behavioral assessments can be unreliable in populations with language barriers or cognitive impairment. Cortical auditory evoked potentials (CAEPs) can serve as a supplementary measurement as they often show strong correlations with SPiN outcomes across diverse hearing profiles.

**Methods:**

Following PRISMA and SWiM guidelines, this systematic review includes studies from PubMed, Web of Science, and Scopus databases that examined the relationship between non-task related CAEPs and SPiN outcomes in adults with normal hearing, SNHL, or cochlear implants.

**Results:**

Sixteen studies were included, encompassing 238 participants with SNHL and 204 participants with normal hearing. Across studies, N1 latency, P2 latency, and N1-P2 amplitude of the onset CAEP and acoustic change complex (ACC) are most consistently correlated with SPiN performance, particularly in sentence-based tests. The mismatch negativity (MMN) showed limited predictive value, as findings varied by age and hearing status. A meta-analysis was not conducted due to methodological heterogeneity.

**Conclusion:**

Onset CAEP and ACC N1 and P2 latencies together with N1-P2 amplitudes particularly demonstrate potential as electrophysiological indicators of SPiN performance. Their clinical utility is promising for populations where behavioral testing can be unreliable, such as CI users or individuals with cognitive or language barriers. However, standardization of protocols and further longitudinal research are needed to validate their application in clinical settings.

**Systematic Review Registration:**

https://www.crd.york.ac.uk/PROSPERO/view/CRD42023404158, identifier PROSPERO (CRD42023404158).

## 1 Introduction

Hearing loss is the most common sensory deficit in humans. According to the World Health Organization (WHO), more than 1.5 billion people suffer from hearing impairment (WHO, 2024). People with hearing loss, from mild impairment onward, frequently struggle with hearing and speech perception in noisy environments, leading to considerable challenges in daily communication and functioning (Billings et al., 2024; Cherry, 1953; Dubno et al., 1984). Moreover, people who suffer from severe to profound hearing loss are at greater risk of experiencing anxiety and depression symptoms compared to the general population (Carlsson et al., 2015). Therefore, intensive monitoring and accurate hearing evaluation are important to help patients receive proper and timely rehabilitation.

To this day, clinicians assess the ability of speech in noise (SPiN) understanding with behavioral SPiN tests. SPiN performance (or abilities) as measured by common SPiN tests can be quantified by using a fixed, adaptive or progressive assessment protocol. Fixed protocols use a constant SNR and report percentage correct scores, reflecting the number of correctly repeated responses. Adaptive protocols vary the SNR based on the listener’s performance to estimate the SNR that yields a target level of accuracy, most often 50% correct. Progressive protocols change the SNR in a predetermined direction regardless of performance and typically derive an SNR50 or convert results to SNR loss (Billings et al., 2024). Behavioral SPiN tests are valuable but can be influenced by factors such as attention and language skills, which may affect reliability. Electrophysiological measures could serve as a complementary tool to support behavioral assessments, particularly in cases where behavioral results are inconclusive or difficult to obtain.

Several types of cortical auditory evoked potentials (CAEPs) are promising as objective measures to evaluate hearing abilities since they can provide insights into auditory processing capabilities (Eggermont and Ponton, 2002). These responses can be broadly categorized into task-related and non-task-related components. Task-related CAEPs, such as the P3 or P300, require active attention and performing a specific task (Picton, 1992). They are often used to study cognitive aspects of auditory processing. In contrast, non-task-related CAEPs can be elicited under passive listening conditions, providing insight into obligatory and automatic auditory processes without requiring behavioral engagement. In certain populations (e.g., young children, individuals with limited language proficiency, or those with cognitive impairments) behavioral SPiN testing can be challenging or yield inconclusive results (Vonck et al., 2022). In such cases, supplementary electrophysiological measures may provide additional insight into auditory processing. Among these, non-task-related CAEPs can be particularly suitable because they do not require active participation yet provide indices of auditory encoding and change detection that can help interpret or complement behavioral results. Thus, for the scope of this review, the focus will be exclusively on the following non-task-related CAEPs and their components: onset CAEP, acoustic change complex (ACC) and mismatch negativity (MMN).

In normal-hearing (NH) adults, the onset CAEP is characterized by a P1-N1-P2 waveform with a first positive peak (P1) occurring around 50 ms after stimulus onset, followed by a negative peak (N1) around 100 ms and another positive peak (P2) around 180 ms (Martin et al., 2007). The onset CAEP response is elicited by the onset of an auditory stimulus after silence. It represents the arrival of sensory information in the auditory cortex and the beginning of central auditory processing. Therefore, this specific response provides insight into the proper encoding of spectral and temporal features of (speech) sounds up to the level of the auditory cortex. The N1 component is generated in the superior temporal lobe, superior temporal gyrus and Heschl’s gyrus (Lightfoot, 2016). The P2 component is suggested to have multiple generators in Heschl’s gyrus as well (Lightfoot, 2016). The P1 component is generally generated in the primary auditory cortex, although it is also thought to have origins in the hippocampus, planum temporale, the lateral temporal cortex, and neocortical areas (Lightfoot, 2016; Howard et al., 2000; Liégeois-Chauvel et al., 1994). The ACC is an obligatory cortical response that shares the characteristic N1-P2 morphology of the onset CAEP but differs in how it is elicited (Martin et al., 2008). Whereas the onset CAEP occurs in response to the beginning of an auditory stimulus, the ACC is evoked by a change within an ongoing sound, such as a shift in frequency or intensity (Martin et al., 2008). Despite these differences in timing and stimulus context, the ACC and onset CAEP components originate from similar cortical regions. The P2 component is primarily associated with the lateral Heschl’s gyrus while the N1 component is generated in regions spanning the lateral Heschl’s gyrus and the supratemporal plane (Cheek and Cone, 2020). Both the onset CAEP as the ACC peak-to-peak amplitudes are noted as N1-P2 amplitudes. However, both N1 and P2 components are also discussed separately in this review. As speech perception depends on accurate discrimination of frequency, intensity, and temporal cues, ACC measures (such as amplitude, latency, and thresholds) are thought to index these underlying neural capacities (Meehan et al., 2025). Evidence from multiple studies shows moderate to strong correlations between ACC responses and behavioral speech perception, supporting its potential as an objective marker of auditory perception relevant to speech understanding (Meehan et al., 2025). A specific component following the onset CAEP or ACC, the N2 component, occurs around 200–300 ms after stimulus onset and is suggested to have generators in Heschl’s gyrus, the supratemporal plane, and the posterior superior temporal gyrus (O'Donnell et al., 1993; Halgren et al., 1992). The Mismatch Negativity (MMN) is a negative-polarity CAEP with a peak latency occurring approximately 100–250 ms after the onset of a deviant stimulus (Giard et al., 1990; Näätänen et al., 2007). It represents the brain’s automatic response to auditory change detection. The MMN is elicited when an infrequent deviant stimulus differs from a repetitive standard stimulus in any discriminable auditory feature (such as frequency, intensity, duration, or location). The MMN response is typically derived from a difference wave, created by subtracting the averaged evoked response to the standard stimulus from the averaged response to the deviant stimulus (Turetsky et al., 2007). This response reflects an automatic, pre-attentive comparison process where incoming auditory information is matched against a sensory memory trace of the standard stimulus. The MMN response is generated by two distinct neural systems: temporal generators in the primary auditory cortex that handle sensory memory comparison, and frontal generators that trigger automatic attention switching when mismatches are detected (Giard et al., 1990). The MMN is considered relevant to SPiN performance because it reflects pre-attentive discrimination, i.e., the brain’s ability to detect acoustic changes automatically. This process supports extraction of speech cues in complex listening environments, and MMN characteristics such as amplitude, latency, and associated oscillatory activity have been linked to individual differences in SPiN (Koerner et al., 2016). As this is the case for NH adults, it should be noted that CAEP amplitudes and latencies are susceptible to listener characteristics such as age and degree or severity of hearing loss (Gürkan et al., 2024). Regarding age, some studies found longer latencies in older adults (Anderer et al., 1996; Tremblay et al., 2004), while others found no difference between young and older populations (Amenedo and Díaz, 1998; Kim et al., 2012). CAEP amplitudes were increased in some studies (Anderer et al., 1996; Ross et al., 2007), while others found decreased amplitudes (Tremblay et al., 2004) or even no effect between young and older participants (Iragui et al., 1993). Regarding SNHL, there are studies reporting prolonged latencies with increasing degree of SNHL (Bertoli et al., 2011; Campbell and Sharma, 2013). CAEP amplitudes were either increased (Campbell and Sharma, 2013; McClannahan et al., 2019; Oates et al., 2002) or diminished (Polen, 1984; Wall et al., 1991). However, studies vary in stimulus and recording parameters, electrode placement, stimulus intensity, and sample size which can all have an effect on the outcome (Gürkan et al., 2024).

Overall, CAEPs assess central auditory processing and have proven effective in evaluating sound processing at the level of the central auditory nervous system (Tomlin and Rance, 2016). Additionally, CAEPs are non-invasive, relatively inexpensive, and mostly unaffected by cultural or educational factors (Meha-Bettison et al., 2018; Peltola et al., 2003). For instance, CAEPs can be used to track auditory development in children, although it remains elusive whether peaks observed during childhood are functionally equivalent to those found in adults (Ponton et al., 2000). However, the literature shows that the P1 peak is present from childhood onward, but decreases with age (Ponton et al., 2000). Furthermore, there is a growing body of evidence that CAEPs are a reliable method to objectively fit a cochlear implant (CI). Unlike electrically-evoked compound action potentials (eCAPs), pulse trains, identical to those used during clinical fittings, can be used as CAEP stimuli (Visram et al., 2015). Moreover, since the response originates from the cortex instead of the auditory nerve, it is more likely to be associated with the actual perception of the stimulus (Visram et al., 2015). Távora-Vieira et al. found that CAEPs can effectively verify CI fitting (Távora-Vieira et al., 2022). The presence of CAEP responses indicated satisfactory CI mapping and led to improvement of speech perception scores, and overall hearing performance.

Building on the role of CAEPs in verifying CI fittings and enhancing speech perception, there is compelling evidence linking CAEP measures to behavioral SPiN testing. This growing body of research suggests that CAEPs can serve as a valuable predictor of how well listeners understand speech in noisy environments (Vonck et al., 2022; Billings et al., 2013; Billings et al., 2015; Han and Dimitrijevic, 2020; Liang et al., 2018; van Heteren et al., 2022; Vonck et al., 2021). Despite the promising correlations between CAEP measures and behavioral SPiN outcomes, there remains considerable variability in the methods used to assess the relationship between SPiN and CAEPs across studies. Several different protocols, stimulus types, and recording techniques are employed, leading to a lack of standardization in the field. Some studies use complex speech stimuli, while others use pure tones or clicks, with varying levels of noise and signal processing strategies.

Given this methodological heterogeneity, the aim of this systematic review is to critically evaluate the existing literature on non-task related CAEPs, their components from N1 onwards, and their utility in predicting SPiN in adult individuals with normal hearing, hearing loss, and those wearing unilateral or bilateral CI. The focus on these non-task related CAEPs is motivated by several clinical considerations. First, these measurements do not require active attention or behavioral responses from subjects, making them particularly valuable for populations where traditional behavioral testing may be challenging or unreliable, such as pediatric populations who may have difficulty maintaining attention during long procedures, or individuals with cognitive impairments who may struggle with task comprehension or execution. Second, non-task related CAEP protocols are essentially language-independent, allowing for standardized assessment across diverse linguistic backgrounds without the confounding effect of language proficiency. Third, the passive nature of these measurements eliminates the need for subject cooperation beyond remaining relatively still and awake, thereby reducing testing burden and increasing clinical feasibility. Finally, although the P1 component is fundamentally part of the P1-N1-P2 onset CAEP complex, it is not examined in the present review. While P1 is a prominent peak in pediatric populations (Ponton et al., 2000), it is comparatively small in adults (Eggermont and Ponton, 2003). The developmental increase in N1 amplitude leads to temporal overlap with the earlier-latency P1, resulting in an apparent reduction in both P1 amplitude and latency across maturation (Eggermont and Ponton, 2003). In children, the robustness of the P1 response has supported its use as a neural correlate of SPiN performance (Eggermont and Ponton, 2003; Xiong et al., 2022). In contrast, because the P1 in adults is markedly reduced and often approaches baseline levels (Billings et al., 2013), there is little empirical work investigating associations between adult obligatory P1 responses and SPiN outcomes. Consequently, in adult populations (and in relation to SPiN performance), the literature focuses predominantly on the N1 response and later auditory evoked potentials. Specifically, the review aims to address the following question: Are non-task related CAEP measurements correlated with and able to estimate SPiN outcomes in adult subjects with normal hearing and varying degrees of sensorineural hearing loss?

## 2 Methods

The protocol of this review has been registered at the PROSPERO International Prospective Register of Systematic Reviews (ID: CRD42023404158) at https://www.crd.york.ac.uk/PROSPERO/. In formulating and drafting this study, the Preferred Reporting Items for Systematic Reviews and Meta-analyses Protocols (PRISMA-P) statement was used as a guideline (Page et al., 2021).

### 2.1 Eligibility criteria

Studies reporting interventions and outcomes of non-task related CAEPs together with behavioral SPiN testing in an adult population presenting with normal hearing thresholds or sensorineural hearing loss (SNHL) were included. Studies investigating CAEPs in patients using CIs were also included. Children or patients with significant neurological disorders as well as animal studies were excluded. Regarding study design, reviews, systematic reviews, and meta-analyses were excluded. Comparison between study populations was not applicable for this review. Included non-task related CAEP measures consisted of the onset CAEP, ACC, and MMN. For onset CAEP and ACC, included components were N1, P2 and N2 latencies and amplitudes, as well as the N1-P2 amplitude. For MMN, latency and amplitude were included. To preserve the targeted focus of this review, any studies that did not specifically and exclusively examined obligatory CAEPs were excluded. Research that primarily investigated related but distinct auditory or cortical processes, such as cortical lateralization, central gain, or sensory gating, was therefore excluded, as these topics fall outside the intended analytical framework.

### 2.2 Search strategy

PubMed, Web of Science, and Scopus were used as databases and searched in the scope of this systematic review. The following search string was used: [(speech intelligibility) OR (speech in noise) OR (hearing in noise) OR (sentence in noise) OR (speech audiometry)] AND [(predict*) OR (prognosis)] AND [(cortical auditory evoked potential*) OR (auditory evoked potential*) OR (event related potential*) OR (N1) OR (P2) OR (N100) OR (EEG) OR (brain)]. The search strategy was adapted for each database. The reference lists of potential sources were screened for additional articles. Only studies in English were included without restrictions regarding publication date. The literature search was conducted from January 2023 to October 2023.

### 2.3 Study selection

After removal of duplicates, titles and abstracts of articles retrieved by database searches were screened by multiple reviewers, such that each record was screened by at least two independent authors (LB + EC and LB + LJ). Articles with titles and abstracts that met the eligibility criteria were subjected to full-text screening by the same authors (LB, EC, LJ). Any disagreements between reviewers were resolved by consensus. If a consensus could not be reached, an additional reviewer (ML) was consulted to make a final decision.

### 2.4 Data extraction

The following data were extracted by all three reviewers (LB, EC, LJ) and can be found in Supplementary Table 1: author, year of publication, characteristics of the study population (number, sex, age, and hearing level), study protocol and methodology, outcome measures, statistical analysis, and results.

Additionally, acquisition parameters of CAEP measurements were extracted as well and are provided in Supplementary Table 2. This entails the equipment, stimulus, number of sweeps, intensity, time window, stimulated ear, and assessment conditions.

Due to the significant diversity observed in the study population and acquisition parameters, conducting a meta-analysis was not feasible. Therefore, results were grouped per type of non-task related CAEP and were reported in a descriptive manner. In order to examine the heterogeneity in results in this systematic review, the Synthesis Without Meta-analysis (SWiM) guideline was used (Campbell et al., 2020).

### 2.5 Risk of bias assessment

All studies that met the inclusion criteria following full-text screening were subjected to a risk of bias and quality assessment using the Quality Assessment Tool for Observational Cohort and Cross-Sectional Studies, designed by the National Institutes of Health (NIH, NIoH, 2021). This checklist assesses potential flaws in observational cohort and cross-sectional studies, including sources of bias and confounding variables. Except for one cohort study, all included studies were prospective cross-sectional studies. The assessment was independently conducted by the same reviewers (LB, EC, LJ) with disagreements resolved through discussion or, if necessary, consultation with an additional reviewer (ML). Given that four items on the checklist were not applicable to the included study designs, each study was scored out of a maximum of 10 points. Based on these scores, studies were rated as ‘good’ (8–10 points), ‘fair’ (5–7 points), or ‘poor’ (1–4 points), acknowledging that some may still be susceptible to bias. Additional information on the criteria that were scored can be found in Supplementary material section 1.

## 3 Results

### 3.1 Study selection

**Figure 1:**
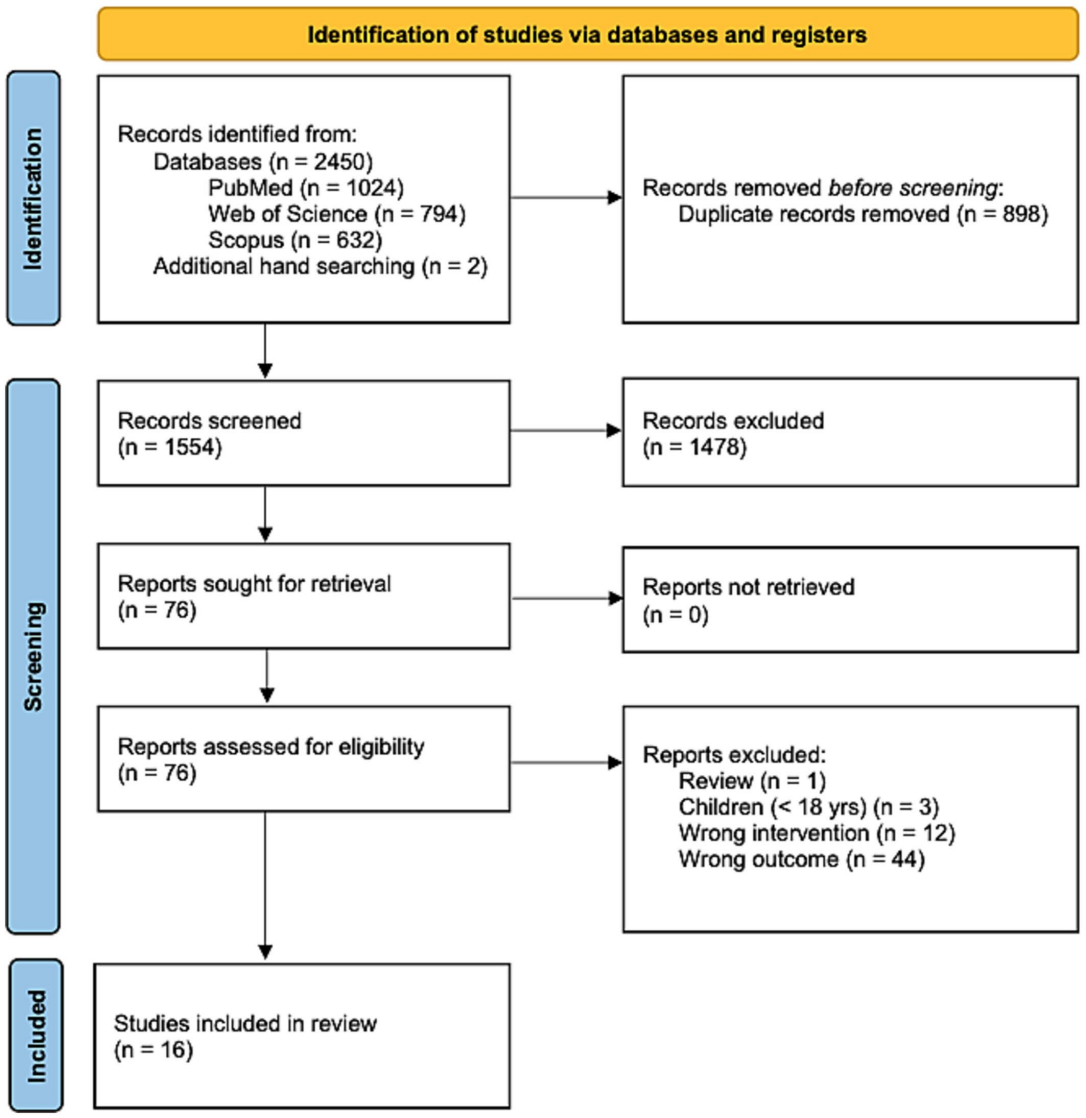
PRISMA flowchart of the study selection procedure.

A total of 2,450 articles were retrieved from the search databases and two articles were retrieved by additional hand searching. After removal of 898 duplicates, the articles were subjected to title and abstract screening. When screening was completed, 1,478 articles were excluded. After full-text screening, 16 articles were included in this systematic review. An overview of the study selection process can be found in the PRISMA flowchart in Figure 1.

### 3.2 Study characteristics

Fifteen cross-sectional studies and one cohort study comparing CAEPs and their correlation with SPiN tests between subjects with hearing loss and/or those with NH were included in this review. All studies combined, 442 subjects were included. Five studies included only NH subjects, while two studies included only individuals with SNHL and nine studies included both NH individuals and those with SNHL. In total, 238 subjects with SNHL were included of whom 165 have at least one cochlear implant. The number of patients with SNHL included per study ranges from 4 to 114. In all studies combined, 204 NH subjects were included. The number of NH subjects per study ranged from 8 to 30. Subjects with SNHL had a mean age of 60.65 years, ranging from 18 to 85 years, while NH participants had a much younger mean age of 41.05 years, ranging from 19 to 78 years. The known proportion of male subjects in the hearing loss group was, on average, 48.74% (ranging from 20 to 76.92%). Among NH participants, the known proportion of male participants was 27.94%, ranging from 23.08 to 58.33%.

The types of non-task related CAEPs measured varied across all included articles. Some papers investigated multiple types of non-task related CAEPs. Nine studies investigated the N1, P2, and/or N2 amplitudes and/or latencies of which two studies focused specifically on onset N1-P2 responses and four studies on the acoustic change complex (ACC). Additionally, three papers investigated MMN amplitude and/or latency.

Across the included articles, there is a notable variation in the SPiN tests used to investigate auditory processing capabilities. Among the reviewed studies, 7 employed fixed SPiN protocols and 11 used adaptive SPiN protocols. Two studies incorporated both a fixed and an adaptive protocol. Fifteen studies used sentences, two articles used words of which one also used digits, while one article used syllables as speech material. Furthermore, multiple articles employed a combination of SPiN tests to explore their correlation with measured CAEPs.

### 3.3 Risk of bias assessment

**Figure 2:**
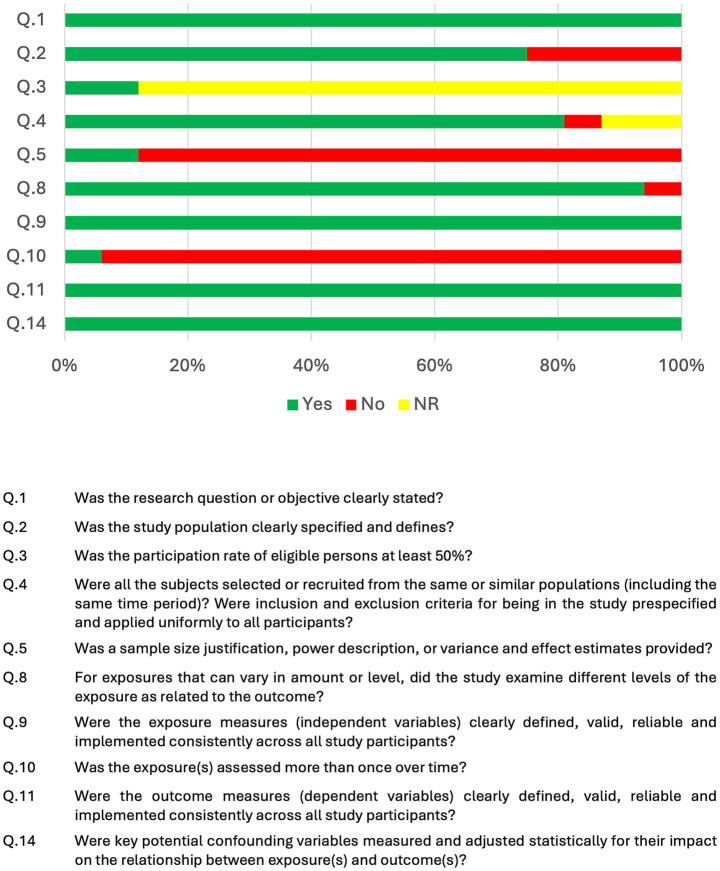
Overview of risk of bias across included studies, assessed using the NIH quality assessment tool for observational cohort and cross-sectional studies (NIH, NIoH, 2021). The bar chart displays the proportion of studies that met each methodological criterion (“Yes”), did not meet the criterion (“No”), or did not report on the criterion (“NR”). Each row corresponds to a specific item from the assessment tool, providing a visual summary of methodological strengths and weaknesses among the included articles.

According to the scores specified in the Methods section, 15 studies were rated as ‘fair’ and four studies received a good quality rating, as summarized in Table 1. In addition, Figure 2 provides an overview of the overall risk of bias per item of the NIH Quality Assessment Tool for Observational Cohort and Cross-Sectional Studies (NIH, NIoH, 2021), illustrating the methodological strengths and weaknesses across all included articles.

**Table 1:** Quality assessment, performed using the quality assessment tool for observational cohort and cross-sectional studies, designed by the National Institutes of Health.

Reference	Score	Quality
Billings et al. (2013)	7	Fair
Campbell and Sharma (2013)	6	Fair
Bidelman and Dexter (2015)	6	Fair
Billings et al. (2015)	8	Good
Brown et al. (2015)	7	Fair
Bidelman and Howell (2016)	6	Fair
Koerner et al. (2016)	6	Fair
Bidelman et al. (2018)	7	Fair
Koerner and Zhang (2018)	7	Fair
Legris et al. (2018)	8	Good
Bidelman et al. (2019)	8	Good
McGuire et al. (2021)	6	Fair
Sohier et al. (2021)	6	Fair
Blankenship et al. (2022)	7	Fair
Vonck et al. (2022)	7	Fair
Berger et al. (2023)	8	Good

This quality assessment tool consists of 14 questions assessing potential flaws in observational cohort and cross-sectional studies, including sources of bias and confounding variables. Some of these questions (numbers 6, 7, 12 and 13) were excluded from scoring as they were not applicable for this systematic review. Consequently, all included articles were given points out of 10 (1–4, poor; 5–7, fair; 8–10, good).

### 3.4 Synthesis of results

**Figure 3:**
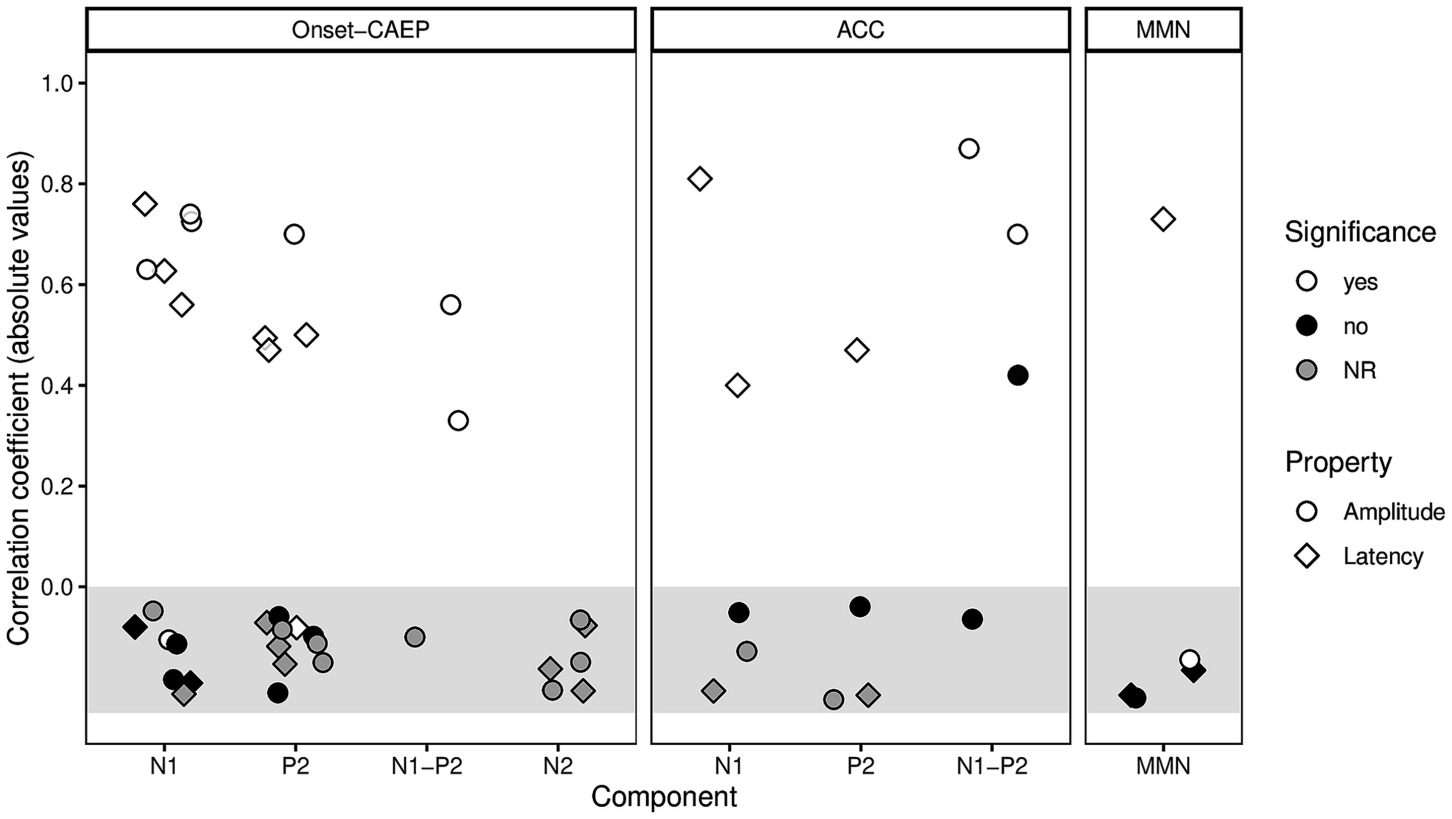
Summary of correlations between non-task related CAEP responses and SPiN outcomes across included studies. The figure is organized into three panels, representing the onset CAEP, ACC, and MMN responses. Within each panel, individual CAEP components are shown with markers indicating the type of measure: circles represent the amplitude and diamonds represent the latency of a component. Correlation coefficients are displayed as absolute values to illustrate the strength of association between each CAEP component and SPiN performance. Marker shading denotes statistical reporting: white for significant correlations, black for non-significant correlations, and gray for components where the significance of correlation results was not reported. The gray zone highlights all components for which the included studies failed to provide any correlation coefficients.

As this review focused on different CAEPs, their components and their relation to SPiN outcomes, the synthesis of results were grouped per type of CAEP (onset CAEP, ACC, and MMN) and reported in a descriptive manner. The relationship between non-task related CAEP components and SPiN outcomes were demonstrated using reported correlation coefficients (i.e., the standardized metric). If correlation coefficients were not available, *p*-values were reported instead. Table 2 and Figure 3 summarize the reporting of correlation outcomes between CAEP components and SPiN measures across included studies.

**Table 2:** Summary of reported correlations between CAEP components and SpiN measures across included studies.

Response type	CAEP component	Corr.	No corr.	NR	Range	Overall direction of effects
Onset CAEP	N1 latency	3	2	1	0.56–0.76	↓ latency, ↑ SPiN
	N1 amplitude	4	2	1	0.63–0.74	↑ amplitude, ↑ SPiN
	P2 latency	3	1	3	0.47–0.76	↓ latency, ↑ SPiN
	P2 amplitude	1	3	3		↑ amplitude, ↑ SPiN
	N2 latency	0	0	3		
	N2 amplitude	0	0	3		
	N1-P2 amplitude	2	0	1	0.33–0.56	↑ amplitude, ↑ SPiN
ACC	N1 latency	2	0	1	0.40–0.81	↓ latency, ↑ SPiN
	N1 amplitude	0	1	1		
	P2 latency	1	0	1		↓ latency, ↑ SPiN
	P2 amplitude	0	1	1		
	N1-P2 amplitude	2	2	0	0.70–0.87	↑ amplitude, ↑ SPiN
MMN	Latency	1	2	0		↓ latency, ↑ SPiN
	Amplitude	1	1	0		↑ amplitude, ↑ SPiN

For each response type (onset CAEP, ACC, MMN) and component (latency/amplitude), the table shows the number of articles reporting a correlation, no correlation, and articles that investigated the component but failed to report the correlation (NR). Ranges of correlation coefficients are shown for components for which at least two correlation coefficients are reported in this review. The overall direction of effects are reported for CAEP components that are correlated with SpiN outcomes.

#### 3.4.1 The correlation between speech perception and the N1, P2, N2 and the N1-P2 complex

Nine of the included articles have investigated the correlation between behavioral speech perception tests and one or more of the CAEPs mentioned above. For the purpose of clarity, articles containing results on the N1, P2, and/or N2 are grouped per behavioral SPiN test. Articles investigating N1-P2 responses are grouped separately.

Two studies specifically looked at the correlation with the Institute of Electrical and Electronic Engineers (IEEE) sentences. Billings et al. (2013) found that the N1 amplitude and latency are the best predictors of SPiN in NH subjects, especially at −5 to 5 dB SNR (*r* = 0.725 and 0.627 respectively; Billings et al., 2013). Billings et al. (2015) investigated the correlation in NH as well as HI subjects (Billings et al., 2015). They found that the N1 and P2 amplitudes and latencies were the best predictors of the SRT (N1 amplitude: *r* = 0.74, N1 latency: *r* = 0.76, P2 amplitude: *r* = −0.80, P2 latency: *r* = 0.76).

Four studies specifically explored the correlation with the Quick Speech in Noise (QuickSIN) test. Campbell et al. (2013) included both NH and hearing impaired (HI) subjects and found a significant correlation between P2 latency and QuickSIN performance (*r* = 0.494, *p* = 0.022) but not for the P2 amplitude (Campbell and Sharma, 2013). HI subjects did not wear hearing aids. Bidelman et al. (2016) included only NH subjects and found that N1 amplitude in the Insula/Broca’s area was significantly associated with SPiN performance for the right ear (*r* = 0.63, *p* = 0.02) but not for the left ear (*r* = 0.23, *p* = 0.47; Bidelman and Howell, 2016). Using a generalized linear mixed-effects framework, Bidelman et al. (2018) evaluated how cortical responses to acoustically degraded speech relate to behavioral SPiN performance (Bidelman et al., 2018). The overall model was significant (*p* = 0.037) and explained a substantial proportion of variance in QuickSIN scores (adjusted R^2^ = 0.81). Examination of individual predictors indicated that N1 amplitudes in the Insula/Broca’s area were a significant neural predictor of SPiN performance (*p* = 0.011). Bidelman et al. (2019) repeated this study in NH and HI participants (Bidelman et al., 2019). This study population showed a significant correlation between P2 latency and QuickSIN performance (*r* = 0.47, *p* = 0.0068), where earlier responses predicted better outcomes. No significant correlations were found for the N1 amplitude or latency.

Three articles specifically investigated the correlation between onset CAEP component morphologies and other SPiN tests. Blankenship et al. (2022) searched for correlations with the AzBio and Bamford-Kowal-Bench (BKB) sentences and the consonant nucleus consonant (CNC) word recognition test in NH and HI participants (Blankenship et al., 2022). The HI participants were CI users. This study only found significant within-frequency correlations (*p* < 0.004). All correlations with across-frequency CAEPs were not significant. Within-frequency correlations revealed a significant negative correlation between AzBio sentences in noise and the N1 latency (*r* = −0.57, *p* ≤ 0.004), and a significant positive relationship between the SRT of the BKB sentences and the N1 latency (*r* = 0.56, *p* ≤ 0.004). Furthermore, they found a significant positive correlation between CNC word scores and the N1-P2 amplitude (*r* = 0.56, *p* ≤ 0.004). In other words, they found that individuals with poorer speech performance had smaller N1-P2 amplitudes and longer N1 latencies. Berger et al. (2023) found a significant correlation between the N1-P2 complex and California consonant test (CCT) accuracy (*r* = 0.33, *p* < 0.001) in HI subjects that were either bilateral, unilateral, or hybrid CI users (Berger et al., 2023). Larger amplitudes predicted better performance in low SNR conditions. Moreover, they found a significant positive correlation between the N1-P2 complex and CNC accuracy (*r* = 0.32, uncorrected *p* = 0.002). Amplitudes did not significantly correlate with AzBio scores (*r* = 0.13, *p* = 0.27). One study of the included articles on N1-P2 responses did not find a significant correlation with the used behavioral SPiN test, the sentences from Marginal Benefit from Acoustic Amplification (MBAA) corpus (Legris et al., 2018). It should be noted that a few studies did not report whether testing was done with or without hearing aids in HI participants (Billings et al., 2015; Bidelman et al., 2019).

#### 3.4.2 The correlation between speech perception and the ACC

Four articles specifically investigated the correlation between behavioral SPiN tests and the ACC. McGuire et al. studied the ACC response and its correlation with the CNC word recognition test and Digits-In-Noise (DIN) test in HI listeners, wearing a CI (McGuire et al., 2021). ACC responses were evoked to 0, 10 and 70% frequency changes in tone stimuli with base frequencies of 0.25, 1, and 4 kHz. They found that the mean N1 latency was negatively correlated with CNC word recognition (*r* = −0.40, *p* < 0.05) and CNC phoneme (*r* = −0.40, *p* < 0.05). The P2 latency was positively correlated with DIN performance (*r* = 0.47, *p* < 0.05). No significant correlations between ACC amplitude and speech outcomes were found in this study. Sohier et al. analyzed the correlation between BKB sentences and the ACC response in both NH and HI listeners, who wore hearing aids during testing (Sohier et al., 2021). ACC responses were evoked to the transition from spectral ripple noise with a phase inversion of 0° to 180°. This study found that SPiN scores were significantly correlated with ACCs (*p* < 0.05). More specifically, participants exhibiting higher ACC amplitudes had better SPiN scores. Additionally, they report that the correlation was the strongest when measured with high-pass filtered SNRs presented at normal conversational level (*r* = 0.70, *p* < 0.05). Vonck et al. measured the ACC at different base frequencies (0.5, 1, 2, and 4 kHz) that increased 12% in frequency, and explored any correlation with speech scores from the Dutch Standardized Sentences by Plomp & Mimpen in NH and HI participants, tested without hearing aids (Vonck et al., 2022). They found a moderate to strong correlation between the SRT and ACC amplitude at each base frequency (*r* = −0.46 to −0.67, *p* < 0.005). Furthermore, SRT was significantly correlated to ACC latency at each base frequency (*r* > 0.48, *p* < 0.004), except at 0.5 kHz (*p* = 0.24). One of the included articles on the ACC did not find a significant correlation (*r* = 0.42, *p* > 0.05) with the behavioral SPiN test, which used an adaptive procedure to determine the SNR at which participants could correctly identify 50% of 12 presented spondees in background noise (Brown et al., 2015). In this study, ACC responses were evoked to vowel changes from /u/ to /j/ and from /j/ to /u/.

#### 3.4.3 The correlation between speech perception and MMN

Three included studies investigated the correlation between MMN and speech perception scores. Two studies by Koerner et al. studied the MMN as predictor of behavioral phoneme change-detection (Koerner et al., 2016; Koerner and Zhang, 2018). In order to evoke the MMN in both studies, the deviant stimuli /da/ and /bu./ (104 trials) needed to be distinguished from the standard /ba/ stimulus (832 trials). Their first study in 2016 included only NH subjects and found that the MMN amplitude in response to /bu./ was the only significant predictor of behavioral sentence-level scores (Koerner et al., 2016). Their study of 2018 had the same protocol, but measurements were done on a study population of NH and HI subjects, tested without hearing aids (Koerner and Zhang, 2018). In this study, they found no significant correlations between MMN amplitude or latency with behavioral sentence-level scores. Bidelman and Dexter (2015) examined MMN latency as a predictor of SPiN performance across two cortical regions (i.e., the Insula/Broca’s area and the superior temporal gyrus (STG)) and analyses were conducted separately for monolingual and bilingual participants. Among monolingual listeners, MMN latency in the STG was not significantly associated with SPiN performance (*r* = 0.24, *p* > 0.05), whereas a strong and significant correlation was observed in the Insula/Broca’s area (*r* = 0.73, *p* < 0.05). In contrast, among bilingual listeners, the pattern was reversed: MMN latency in the STG showed a significant positive correlation with SPiN performance (*r* = 0.69, *p* < 0.05), while the association in the Insula/Broca’s area was not significant (*r* = 0.22, *p* > 0.05). In this study, the MMN was evoked by detecting the deviant /tᴐt/ stimulus (120 trials) from the standard /tat/ stimulus (680 trials).

## 4 Discussion

This systematic review synthesized evidence on the relationship between non-task related CAEPs and SPiN outcomes across adults with normal hearing, SNHL, including CI-users. To our knowledge, this is the first paper to systematically review non-task related CAEPs and their relationship to SPiN scores. A meta-analysis was not feasible, due to heterogeneity in the included study populations as well as the used speech materials and acquisition parameters. Therefore, findings were integrated using the SWiM guideline. Overall, the most consistent associations with SPiN performance were observed for N1 latency, P2 latency, and N1-P2 amplitude, derived primarily from onset CAEP measures (reflecting their greater representation in the literature) and supported by findings from the ACC, whereas MMN components showed limited and less consistent relationships (Figure 3). Notably, several included studies did not report correlation values for all components examined, which constrains interpretability and may indicate reporting bias.

Both the N1 and P2 latency as well as the N1-P2 amplitude of both the onset CAEP and ACC correlated in a specific direction with the SPiN tests. Shorter latency values and larger N1-P2 amplitudes correlated with better SPiN performance (i.e., higher percentage correct score or lower SRT values). These effects appear to be independent of the choice of CAEP stimulus or SPiN test, although most correlations were found with sentence-based tests. Two articles did not find a correlation between N1 and P2 parameters and sentence-based SPiN test (Berger et al., 2023; Legris et al., 2018). Compared to other included studies that reported significant correlations, several methodological and population-level differences may explain these findings. Both studies used sound-field auditory stimulation, which can introduce variability in sound presentation and cortical response timing, particularly in CI-users (Dennison et al., 2022). Legris et al. (2018) focused on single-sided deafness (SSD) CI users, a population with asymmetric auditory input and potential cortical reorganization, while Berger et al. (2023) included a large and heterogenous CI population, increasing inter-subject variability. Additionally, the latter used high SNRs (+7 and +13 dB), which may not sufficiently challenge the auditory system in order to correlate the results to behavioral SPiN tests.

Two of the included articles used tone-based stimuli of similar frequencies to record CAEPs and correlated these responses with sentence-based behavioral SPiN tests (Vonck et al., 2022; Blankenship et al., 2022), in contrast to other included studies that employed speech syllables or sentences (Koerner et al., 2016; Billings et al., 2013; Billings et al., 2015; Bidelman and Howell, 2016; Bidelman et al., 2018; Bidelman et al., 2019; Berger et al., 2023; Legris et al., 2018; Brown et al., 2015; Koerner and Zhang, 2018; Bidelman and Dexter, 2015; Papesh et al., 2017; Slugocki et al., 2022). Despite differences in the number of electrodes used during recording, both studies found similar results. Significant correlations were observed between N1 latency, N1-P2 amplitude, and behavioral SPiN tests. Specifically, individuals with poorer speech performance exhibited smaller N1-P2 amplitudes and longer N1 latencies. Both studies involved subjects of similar age. Blankenship et al. (2022) focused on CI-users, while Vonck et al. (2022) studied subjects without CI. Additionally, McGuire et al. (2021) investigated CI-users and found a significant correlation with N1 latency, using CNC scores to correlate with ACC responses that were evoked by the same change stimuli that Vonck et al. used. The consistent findings across these studies suggest that EEG recordings to tone-based stimuli may be suited as a reliable, supplementary measurement to evaluate SPiN ability in NH individuals, those with hearing impairment, and CI-users. More importantly, as these CAEPs evoked to language-independent tone-based stimuli correlate to behavioral SPiN tests, it could be used to assess speech perception in individuals that do not speak the language of the behavioral test but also in individuals with cognitive impairment.

Only three articles investigated the MMN amplitude and latency in relation to SPiN performance. Bidelman and Dexter (2015) and Koerner et al. (2016) both studied NH subjects of the comparable age, using sentence-based behavioral tests with percentage correct score as the outcome. However, their results diverged. Bidelman et al. reported that MMN latency showed the strongest association with SPiN performance, whereas Koerner et al. found that MMN amplitude was the primary correlate. One methodological difference between the studies is the stimulus presentation level used to elicit the MMN. Bidelman et al. used 80 dB SPL while Koerner et al. (2016) used 60 dB SPL. This difference may have contributed to the discrepancy in which MMN component was most predictive. The third MMN study, also by Koerner and Zhang (2018), used the same EEG recording protocol and behavioral tests as their previous work (Koerner et al., 2016), but included both NH subjects and those with SNHL who were older than those in the other MMN studies. No correlations with behavioral SPiN scores were found. These mixed results suggest that the relationship between MMN features and SPiN perception may be sensitive to methodological factors such as stimulus level, as well as listener characteristics, including age and hearing status.

### 4.1 Clinical implications

Electrophysiological hearing assessments are of interest when behavioral audiometric results yield questionable or unexpected outcomes. For example, it remains a challenge to accurately assess behavioral speech perception in patients with cognitive impairment, language barriers, or in young children. For these populations, it is not only difficult to assess their hearing performance and speech perception ability, but also to fit hearing aids and determine cochlear implant candidacy. An electrophysiological indicator of speech perception performance could provide a valuable addition to existing behavioral measures.

Most of the included articles in this review have searched for a correlation between onset CAEP outcome measures and behavioral SPiN outcomes. Vonck et al. (2022) states that the onset CAEP might not be a good predictor of SPiN as it does not provide information on advanced auditory processing. The weak correlation between SPiN and onset CAEP in response to a pure tone has been documented in other studies as well (Brown et al., 2015; Billings et al., 2009; Lammers et al., 2015). While this reasoning is plausible, this review contradicts that statement showing many (strong) correlations between N1 latency, P2 latency, and N1-P2 amplitude of the onset CAEP with SPiN outcomes. However, a correlation between two variables does not necessarily mean that one variable can predict the other. The ACC might be an alternative for clinical application as an indicator of auditory performance such as speech perception (Meehan et al., 2025). However, the choice of evoking stimuli, outcome measures, and behavioral SPiN measures still play an important role, as they can influence the degree of correlation between the ACC response and the behavioral SPiN test. The onset CAEP and ACC present several clinical benefits compared to the MMN response. The ACC typically exhibits larger amplitudes and better SNR, making it easier to detect and interpret in clinical settings (Martin and Boothroyd, 1999). Furthermore, onset CAEP and ACC responses can be recorded in a fairly short time (depending on the protocol), which is particularly advantageous when working with populations where time and cooperation are limited, such as young children or individuals with cognitive impairment (Martin and Boothroyd, 1999; Guyonnet-Hencke et al., 2025). Unlike the MMN response, the onset CAEP and ACC demonstrate excellent consistency across repeated measurements (Martin and Boothroyd, 1999; Picton, 1995; Friesen and Tremblay, 2006; Tremblay et al., 2006; Tremblay et al., 2003; Tremblay et al., 2003). Additionally, while informative of auditory discrimination, the MMN requires longer recording times and more complex paradigms, limiting its feasibility in routine clinical settings. The ACC shows particular promise in pediatric populations, as it can be recorded in children as young as 2–3 months of age, providing an electrophysiological measure when behavioral assessments are unreliable (Ching et al., 2023; Strahm et al., 2022). However, CAEP and ACC morphologies change with age due to auditory system maturation, requiring careful interpretation in younger populations (Strahm et al., 2022; Wunderlich and Cone-Wesson, 2006; Sharma et al., 1997). These developmental changes may provide a valuable way to track SPiN perception from childhood through adulthood. CAEP and ACC recordings are clinically feasible in children, but similar to behavioral testing, protocols are often longer than in adults because it can be challenging to keep children still, alert, and quiet (Meehan et al., 2024). Despite these considerations, CAEPs remain a practical and robust electrophysiological tool as an indication for auditory performance and SPiN.

### 4.2 Limitations and directions for future research

Given the heterogeneity in patient populations, behavioral SPiN tests and acquisition parameters of the different CAEPs, it is rather difficult to determine which CAEP and which SPiN outcome are the ideal combination to assess one’s ability to understand speech in an electrophysiological manner. Therefore, there is a need for standardization to facilitate more consistent and comparable results. Before CAEPs can be considered to be clinically implemented to assess SPiN abilities, validation studies are required. Studies with large sample sizes are needed to validate previously reported findings in both NH populations and those with varying degrees of hearing loss. Longitudinal studies are needed to explore the predictive value of CAEPs over time and across different populations. Additionally, although CAEPs provide additional insights in speech understanding abilities, their measurements can be rather long. Therefore, there might be a need to look into shortening the recording protocol in order to facilitate their use in clinical settings. Moreover, not all studies transparently report correlation coefficients when investigating correlation between CAEP components and SPiN outcomes. *p*-values are most often reported, but these are not a reliable metric to evaluate correlation.

While CAEPs are referred to as a relatively inexpensive method to assess auditory and speech perception abilities, it should be noted that these tests remain more expensive than standard behavioral SPiN tests. Their implementation, interpretation, and analysis require trained clinicians and can be time-consuming, particularly when electrode caps are used, as these involve more preparation compared to simplified EEG setups. Additionally, the cost of EEG equipment is substantial, which may limit accessibility in routine practice. This contrasts with behavioral SPiN tests that are already available, widely accessible and easier to carry out by trained audiologists. Although literature shows that CAEPs can be a valuable tool for evaluating hearing, these practical constraints should be considered when interpreting findings and planning future applications.

It is imperative to note that, even though this review reports studies that find correlations between CAEPs and speech perception outcomes, normative data in order to use CAEPs as a diagnostic tool are lacking. Moreover, it would be challenging to determine such normative values as CAEP responses and their morphology rely on a number of factors (age, hearing status, cognitive abilities etc.). Regarding future clinical testing, Koravand et al. suggested reducing the number of EEG recording channels and developing normative data with a simplified EEG electrode setup (Koravand et al., 2012). Only a few of the included articles in this review, used a simplified electrode setup (Vonck et al., 2022; Sohier et al., 2021; Brown et al., 2015; Slugocki et al., 2022).

In addition to these methodological considerations, the overall quality and risk of bias among the included studies should be taken into account when interpreting the findings of this review. As illustrated in Figure 2, the NIH Quality Assessment Tool for Observational Cohort and Cross-Sectional Studies revealed that most studies performed well in domains such as clearly stated research questions and well-defined study populations. However, several areas of methodological weakness were identified. Notably, many studies did not report a sample size justification or power calculation. Furthermore, repeated assessment of exposures over time was infrequently performed. These sources of bias may limit the reliability and generalizability of the current evidence, underscoring the importance of rigorous study design and transparent reporting in the future. Moreover, transparent reporting of correlation coefficients in studies examining the relationship between CAEP components and SPiN outcomes was often lacking. Many studies predominantly reported *p*-values, which are not a reliable metric for evaluating the strength or direction of correlations. In this review, a substantial proportion of the included articles did not transparently report correlation coefficients, particularly when no significant association was found (Figure 3). This pattern suggests potential reporting and publication bias against negative results, which hampers quantitative synthesis and may inflate the apparent consistency of findings across studies. To support robust interpretation and enable meaningful comparisons, future research should adopt more transparent reporting practices by consistently reporting all correlation coefficients, regardless of statistical significance.

## 5 Conclusion

Despite heterogeneity that precluded meta-analysis, the converging evidence indicates that N1 and P2 latencies and N1-P2 amplitude from onset CAEP and ACC show the clearest and most consistent associations with SPiN, especially on sentence-based measures, whereas MMN correlations are less robust and appear population-dependent. These findings support the use of CAEPs as an electrophysiological index of auditory processing that can enhance the interpretation of behavioral SPiN tests, with particular promise in settings where behavioral assessments are unreliable or infeasible. However, further research is still needed to standardize protocols and, more importantly, validate their clinical utility.

## Data Availability

The original contributions presented in the study are included in the article/Supplementary material, further inquiries can be directed to the corresponding author.
