# Supplementary material for: Speech in noise prediction by use of cortical auditory evoked potentials in normal hearing and sensorineural hearing loss: a systematic review
Source: Front Neurosci. 2026 Apr 9;20:1713335. doi: 10.3389/fnins.2026.1713335 (PMC13102654; doi:10.3389/fnins.2026.1713335)
Supplement: Supplementary file 1 [file Supplementary_file_1.docx]

Supplementary Material

# Items of Quality Assessment

To assess the quality of the included studies, the Quality Assessment Tool for Observational Cohort and Cross-Sectional Studies, designed by the National Institutes of Health was used (23). The quality assessment tool consists of 14 questions that can be answered with yes, no or other (cannot determine (CD), not reported (NR) or not applicable (NA)). Questions that were not applicable in this systematic review were not counted negatively towards quality rating. This was the case for questions 6, 7, 12 and 13. Therefore, all included articles were given points out of 10 for the quality assessment.

1. Was the research question or objective in this paper clearly stated?
2. Was the study population clearly specified and defined?
3. Was the participation rate of eligible persons at least 50%?
4. Were all the subjects selected or recruited from the same or similar populations (including the same time period)? Were inclusion and exclusion criteria for being in the study prespecified and applied uniformly to all participants?
5. Was a sample size justification, power description, or variance and effect estimates provided?
6. For the analysis in this paper, were the exposure(s) of interest measured prior to the outcome(s) being measured?
7. Was the timeframe sufficient so that one could reasonably expect to see an association between exposure and outcome if it existed?
8. For exposures that can vary in amount or level, did the study examine different levels of the exposure as related to the outcome (e.g., categories of exposure, or exposure measured as continuous variable)?
9. Were the exposure measures (independent variables) clearly defined, valid, reliable and implemented consistently across all study participants?
10. Was the exposure(s) assessed more than once over time?
11. Were the outcome measures (dependent variables) clearly defined, valid, reliable and implemented consistently across all study participants?
12. Were the outcome assessors blinded to the exposure status of the participants?
13. Was lost to follow-up after baseline 20% or less?
14. Were key potential confounding variables measured and adjusted statistically for their impact on the relationship between exposure(s) and outcome(s)?

# Supplementary Figures and Tables

## Supplementary Tables

Supplementary Table 1. Data extraction of study parameters and results.

| **Reference (first author, year, reference number)** | **Subjects** | **Controls** | **Outcome measures** | **Results** |
| --- | --- | --- | --- | --- |
| Billings et al. (2013) (40) |  | N = 15  7 ♂, 8 ♀  Mean age: 27.6 yrs (range: 23-34)  PTA range: ≤ 25 dB HL | SPiN test: IEEE sentences  Cortical measures: amplitude and latency of N1, P2, N2 | N1 amplitude and latency are the best predictors, especially at -5 to +5 dB SNR.  Pearson correlation coefficients between N1 amplitude/latency and SPiN were 0.725 and 0.627, respectively. |
| Campbell et al. (2013) (28) | N = 9  ♂/♀ NR  Mean age ± SD: 56.9 ± 6.2 yrs | N = 8  ♂/♀ NR  Mean age ± SD: 50.5 ± 8.9 yrs | SPiN test: QuickSIN  Cortical measures: amplitude and latency of P2 | P2 latency showed a significant positive correlation with speech performance (*r* = 0.494, *p* = 0.022): increase in P2 latency is associated with greater difficulty with SPiN.  No significant correlation found between SPiN outcome and P2 amplitude (correlation coefficient not reported). |
| Bidelman et al. (2015) (59) |  | N = 20, monolinguals vs bilinguals  ♂/♀ NR  Age range: 21-34  PTA range: ≤ 25 dB HL | SPiN: QuickSIN test  Cortical measures: amplitude and latency of MMN | Only MMN latency is significantly correlated with SPiN recognition in normal-hearing mono- and bilingual subjects. For monolingual subjects, MMN latency is correlated to SPiN in Ins/Broca’s area (*r* = 0.73, *p* < 0.05), but not in STG area (*r* = 0.24, *p* > 0.05). For bilingual subjects, correlation between MMN latency and SPiN was significant in STG (*r* = 0.69, *p* < 0.05), but not in Ins/Broca’s area (*r* = 0.22, *p* > 0.05). |
| Billings et al. (2015) (41) | N = 15  9 ♂, 6 ♀  Mean age: 72.8 yrs (range: 63-84)  - OHI listeners  Mean PTA threshold ± SD: 52.7 ± 8.6 | N = 30  13 ♂, 17 ♀  - YNH listeners (N = 15)  Mean age: 27.6 yrs (range: 23-34)  PTA range: ≤ 25 dB HL  - ONH listeners (N = 15)  Mean age: 69.4 yrs (range: 60-78)  Mean PTA threshold ± SD: 30.2 ± 10.3 dB HL | SPiN: IEEE sentences  Cortical measures: amplitude and latency of N1, P2 and N2 | N1 and P2 latencies and amplitudes are the best predictors.  Best correlation coefficients for:   - N1 amplitude: 0.74 - N1 latency: 0.76 - P2 amplitude: -0.80 - P2 latency: 0.76 |
| Brown et al. (2015) (57) | N = 10, with a CI  2 ♂, 8 ♀  Mean age ± SD: 58 ± 13.1 yrs (range: 29-78) | N = 10  3 ♂, 7 ♀  Mean age ± SD: 29 ± 7.53 yrs (range: 21-43)  PTA range: ≤ 20 dB HL | SPiN: spondees (two long syllables)  Cortical measures: amplitude and latency of N1 and P2 | No significant correlation (*r* = 0.42) was found between N1-P2 amplitude and performance. |
| Bidelman et al. (2016) (49) |  | N = 12  ♂/♀ NR  Mean age ± SD: 24.7 ± 2.7  PTA range: ≤ 25 dB HL | SPiN: QuickSIN test  Cortical measures: amplitude and latency of N1, P2 and N2 | N1 amplitude in the Insula/Broca’s area was significantly associated with SPiN performance for the right ear (*r* = 0.63, *p* = 0.02) but not for the left ear (*r* = 0.23, *p* = 0.47). |
| Koerner et al. (2016) (19) |  | N = 15  5 ♂, 10 ♀  Mean age: 22.6 yrs (range: 19-32)  PTA range: ≤ 25 dB HL | SPiN: IEEE sentences  Cortical measurements: amplitude, latency of MMN | The MMN amplitude in response to /bu/ was the only significant predictor of behavioral performance (*p* < 0.05).  Correlation coefficients between MMN components and SPiN not reported. |
| Bidelman et al. (2018) (50) |  | N = 11  ♂/♀ NR  Mean age ± SD: 24.7 ± 2.7 yrs  PTA range: < 25 dB HL | SPiN: QuickSIN test  Cortical measurements: N1 amplitude | The overall linear mixed effects model was significant (*p* = 0.037) and explained a substantial proportion of variance in QuickSIN scores (adjusted R^2^ = 0.81). Examination of individual predictors indicated that N1 amplitudes in the Insula/Broca’s area were a significant neural predictor of SPiN performance (*p* = 0.011).  Correlation coefficients between N1 amplitudes and SPiN not reported. |
| Koerner et al. (2018) (58) | N = 4  2 ♂, 2 ♀  Mean age ± SD: 66.75 ± 4.0 yrs (range: 62-71) | N = 14  5 ♂, 9 ♀  Mean age ± SD: 60.8 ± 8.8 yrs (range: 40-69) | SPiN: IEEE sentences  Cortical measures: amplitude and latency of MMN | No correlations were found between MMN amplitude or latency with behavioral sentence-level scores.  Correlation coefficients between MMN components and SPiN not reported. |
| Legris et al. (2018) (54) | N = 9, SSD subjects with at least one year of experience with their CI  4 ♂, 5 ♀  Mean age ± SD: 60 ± 7 yrs  PTA range: > 70 dB HL | N = 8  2 ♂, 6 ♀  Mean age ± SD: 54 ± 3.2 yrs (range: 50-60)  PTA range: ≤ 25 dB HL | SPiN: Sentences from Marginal Benefit from Acoustic Amplification (MBAA) corpus  Cortical measures: amplitude and latency of N1 and P2 | No correlation was found between speech perception and cortical measurements. Correlation coefficients not reported. |
| Bidelman et al. (2019) (51) | N = 19  11 ♂, 8 ♀  Mean age ± SD: 70.4 ± 4.9 yrs  Mean PTA threshold ± SD: 26.4 ± 7.1 dB HL | N = 13  5 ♂, 8 ♀  Mean age ± SD: 66.2 ± 6.1 yrs  Mean PTA threshold ± SD: 15.3 ± 3.27 dB HL | SPiN: QuickSIN test  Cortical measures: amplitude and latency of N1 and P2 | P2 latency (in noise) correlated with QuickSIN scores (*r* = 0.47; *p* = 0.0068), where earlier responses predicted better performance on the QuickSIN test. |
| McGuire et al. (2021) (55) | N = 21, all CI-users (9 unilateral, 12 bilateral)  12 ♂, 9 ♀  Mean age ± SD: 57.85 ± 14.61 yrs (range: 20-83) |  | SPiN: CNC Word Recognition Test, AzBio sentences and DIN  Cortical measures: amplitude and latency of N1 and P2 | Peak latencies were correlated with speech scores.  Mean N1 latency was negatively correlated with CNC scores *r* = -0.40, *p* < 0.05).  Mean P2 latency was positively correlated with DIN performance (*r* = 0.47, *p* < 0.05). |
| Sohier et al. (2021) (56) | N = 13  10 ♂, 3 ♀  Mean age ± SD: 68.1 ± 15 yrs (range: 36-81) | N = 13  3 ♂, 10 ♀  Mean age ± SD: 27 ± 9.6 yrs (range: 19-55)  PTA range: < 20 dB HL | SPiN: Bamford-Kowal-Bench (BKB)-like sentences  Cortical measures: amplitude of the onset-CAEP and ACC | Speech perception was significantly correlated with ACCs in different presentation conditions: HP20NL (*r* = 0.70, *p* < 0.01), HP20LL (*r* = 0.60, *p* < 0.01), LP20NL (*r* = 0.44, *p* < 0.05), and LP20LL (*r* = 0.51, *p* < 0.05), but not onset responses.  The strongest correlation was found between speech perception in noise and ACCs measured with high-pass filtered SNRs presented at normal conversational level (*r* = 0.70, *p* < 0.01). |
| Blankenship et al. (2022) (52) | N = 11, all CI-users  5 ♂, 6 ♀  Mean age: 50.2 yrs (range: 25.2-68.3)  PTA range: 15-45 (with CI) | N = 11  ♂/♀ NR  Mean age: 49 yrs (range: 24.7-68.5)  PTA range: ≤ 25 dB HL | SPiN: AzBio sentences and BKB-sentences  Cortical measures: amplitude and latency of N1 and P2 | Within frequency correlations revealed a significant negative correlation between AzBio-Noise and N1 latency (*r* = -0.57, *p* ≤ 0.004), a significant positive relationship between the BKB-SIN SNR50 and N1 latency (*r* = 0.56, *p* ≤ 0.004), and a significant positive relationship between CNC word scores and N1-P2 amplitude (*r* = 0.56, *p* ≤ 0.004).  Individuals with poorer speech performance had a smaller N1-P2 amplitude and longer N1 latency.  None of the correlations reached significance for across-frequency CAEPs. |
| Vonck et al. (2022) (8) | N = 13  6 ♂, 7 ♀  Mean age ± SD: 43.5 ± 15.4 yrs (range: 20-66)  PTA range: 5-70 dB HL at 0.5 kHz; 40-90 dB HL at 4 kHz | N = 24  14 ♂, 10 ♀  Mean age ± SD: 41.6 ± 13.9 yrs (range: 23-60) | SPiN: Dutch standardized sentences by Plomp and Mimpen (1979)  Cortical measures: amplitude and latency of N1-P2 (onset-CAEP and ACC) | Multiple linear regression analysis revealed that average HL and average ACC latency explained 81% of the total variance in SRT (*r* = 0.81, *p* < 0.001).  The strongest multiple linear regression model was obtained by averaging over 1, 2 and 4 kHz and explained 87% of the total variance in SRT (*r* = 0.87, *p* < 0.001).  There was a moderate to strong correlation between SRT and ACC amplitude at each base frequency (*r* = -0.46 to -0.67, *p* < 0.05).  SRT was significantly correlated to ACC latency at 1, 2 and 4 kHz (*r* > 0.48, *p* < 0.004), but not at 0.5 kHz (*p* = 0.24). |
| Berger et al. (2023) (53) | N = 114 (5 bilateral, 72 unilateral, 87 hybrid CI-users)  55 ♂, 59 ♀  Mean age ± SD: 62.6 ± 13.5 yrs (range: 18-85)  Mean PTA threshold ± SD: 59.4 ± 20.5 dB HL |  | SPiN: California Consonant Test and AzBio sentences  Cortical measures: amplitude of N1-P2 | Significant correlation between N1-P2 complex and CCT accuracy (*r* = 0.33, *p* < 0.001), with larger amplitudes predicting better performance in low SNR condition. Other predictor variables did not exhibit significant correlations.  N1-P2 amplitudes did not significantly correlate with AzBio accuracy (*r* = 0.13, *p* = 0.27). |

Supplementary Table 2. Summary of the used CAEP equipment and acquisition parameters.

| **Reference (first author, year, reference number)** | **Equipment** | **Software** | **Electrode montage (number referring to number of electrodes)** | **Stimulus type** | **SNR levels (dB)** | **Headphones/inserts/free-field** | **Level** | **Sampling rate (Hz)** | **Time window (ms)** | **Sweeps** | **Artifact rejection threshold** | **Filtering (Hz) (high-pass HP; low-pass LP; band-pass BP)** | **Stimulated ear** | **Condition** |
| --- | --- | --- | --- | --- | --- | --- | --- | --- | --- | --- | --- | --- | --- | --- |
| Billings et al. (2013) (40) | Compumedics Neuroscan 4.5 | Neuroscan software (Neuroscan, Inc 2007) | 64-electrode cap | /ba/ | -10-35 (noise fixed) | Inserts | 50, 60, 70 & 80 (dBC) | 1000 | 800 | 150 | ± 70 µV | 100 (LP) | Right |  |
| Campbell et al. (2013) (28) | Electrical Geodesic, Inc | Net Station 4 (Electrical Geodesic, Inc) | 128-channel electrode net | /ba/ | Clean | Sound-field | 65 dB HL | 1000 | 692 | 1200 | ± 100 µV | 0.1-200 (BP) | Left and right | Allowed to watch muted, subtitled movie |
| Bidelman et al. (2015) (59) | TDT RP2 (Tucker-Davis Technologies) | NR | 64-sintered Ag/AgCl elektrodes | /tat/ (standard, 680 trials) and /tᴐt/ (deviant, 120 trials) | +10, 0 and -5 (target fixed) | Inserts | 80 dB SPL | 500 | 800 | NR | NR | NR | Left and right | Allowed to watch muted, subtitled movie |
| Billings et al. (2015) (41) | Compumedics Neuroscan Stim2/Scan 4.5 | Neuroscan software (Neuroscan Inc 2007) | 64-electrode cap | /ba/ | -10-35 (noise fixed) | Inserts | 50, 60, 70, & 80 (dBC) | 1000 | 1900 | 150 | ± 70 µV | 100 (LP) | Right | Allowed to watch muted, subtitled movie |
| Brown et al. (2015) (57) | National Instruments Data Acquisition Board (DAQ Card-6062E) | MATLAB, Sound Designer Software, LabVIEW software | Cz(+) to Mc (-), Tc (+) to Mc (-), Fpz (+) to Mc (-), Cz (+) to Oz (-), Tc (+) to Oz (-), and Fpz (+) to Oz (-) | Vowel change from /u/ to /i/ and vowel change from /i/ to /u/ | NR | Sound-field | 70 dB SPL | 1000 | NR | 200 | ± 0.8 mV | 1-30 (BP) | Control: random  CI-group: implanted ear (unilateral) | Stay awake, allowed to read or watch captioned videos |
| Bidelman et al. (2016) (49) | TDT RP2 (Tucker-Davis Technologies) | MATLAB | 64 sintered Ag/AgCl electrodes | /ama/ | Clean, +5 and +10 (target fixed) | Inserts | 81 dB SPL | 5000 | NR | 2000 | NR | 1.5-20 (BP) | Left and right | Allowed to watch muted, subtitled movie |
| Koerner et al. (2016) (19) | Advanced Neuro Technology EEG system | BESA 6.0 | 64-electrode cap Ag/AgCl | /ba/ (standard, 832 trials)  /da/ and /bu/ (deviant, 104 trials) | Clean and -3 (target fixed) | Inserts | 60 dB SPL | 512 | 100-300 | 120 | ± 50 µV | 0.5-40 (BP) | Left and right |  |
| Bidelman et al. (2018) (50) | Compumedics Neuroscan | Curry 7 software | 64 sintered Ag/AgCl electrodes | /ama/ | Clean, +5 and +10 (target fixed) | Inserts | 81 dB SPL | 5000 | 750 | 2000 | NR | 0.5-20 (BP) | Left and right |  |
| Koerner et al. (2018) (58) | Advanced Neuro Technology EEG Systems | BESA | 64-channel Ag/AgCl electrode WaveGuard cap | /ba/ (standard, 832 trials)  /da/ and /bu/ (deviant, 104 trials) | Clean and -3 (target fixed) | Inserts | 70 dB SPL | 512 | 800 | 120 | ± 50 µV | 0.5-40 (BP) | Left and right |  |
| Legris et al. (2018) (54) | Compumedics System Neuroscan EEG System | Curry 7 software | 64 electrodes (61 corrected for CI) | /ba/ | NR | Sound-field | 70 dB SPL | 500 | 600 | 692-748 | NR | 0.3-70 (BP) | Left and right |  |
| Bidelman et al. (2019) (51) | TDT RP2 (Tucker-Davis Technologies) | MATLAB  BESA | 32 electrodes | /ba/, /pa/ (standard, 3000 trials)  /ta/ (deviant, 210 trials) | Clean, +10 (target fixed) | Inserts | 75 dB SPL | 20000 | 210 | 2070 per block (6210 in total) | NR | 1-30 (BP) | Left and right |  |
| McGuire et al. (2021) (55) | Neuroscan EEG system (Compumedics Neuroscan) | MATLAB  EEGLAB | 40 electrodes | Frequency change (0, 10 and 70%) in tone stimuli (base frequencies: 0.25, 1, and 4 kHz) | Clean | Sound-field | 70 dB SPL | 1000 | 1100 | 400 | NR | 0.1-100 (BP) | Left and right | Depending on patient, CI-ears were tested |
| Sohier et al. (2021) (56) | HEARLab system | MATLAB | FCz, M1, M2, Fz | Onset of spectral ripple noise (SRN) and transition between SRN with phase inversion from 0° to 180° | Clean | Sound-field | 65 dB SPL | NR | 900 | 120 | ± 60 µV | NR | Left and right |  |
| Blankenship et al. (2022) (52) | Neuroscan recording system | Audacity software (version 1.2.5)  Neuroscan software (version 4.3)  MATLAB EEGLAB 13.6.5b | 40-channel Neuroscan Quick-Cap with contralateral earlobe as reference.  1-3 electrodes around coil not used | Pure-tone (1 and 2 kHz, within and across frequency gap detection) | Clean | Sound-field | MCL | 1000 | NR | NH: 200  CI: 400 | NR | 0.1-30 (BP) | Unilateral |  |
| Vonck et al. (2022) (8) | Medelec Synergy T-10 Evoked Potential System | MATLAB | Ag/AgCl electrodes on Cz, A1/A2, Fz plus above and below the eye | Frequency change (12%) in tone stimuli (base frequencies: 1, 2, and 4 kHz) | Clean | Headphones | 75 dB SPL  MCL (max 90 dB SPL) | 50000 | 1000 | 100 | ± 100 µV | 0.01-100 (BP) | Monaurally, better ear |  |
| Berger et al. (2023) (53) | BioSemi Active Two EEG system | MATLAB: Psychtoolbox 3;  BioSemi ActiView | 64-electrode cap | California Consonant Test | +7 and +13 (target fixed) | Sound-field | 70 dB SPL | 2048 | 2500 | 100 | NR | 1-500 (BP) | Left and right |  |
